# High Efficiency Removal Performance of Tetracycline by Magnetic CoFe_2_O_4_/NaBiO_3_ Photocatalytic Synergistic Persulfate Technology

**DOI:** 10.3390/molecules29174055

**Published:** 2024-08-27

**Authors:** Juanxiang Zhang, Shengnan Zhang, Xiuqi Bian, Yaoshan Yin, Weixiong Huang, Chong Liu, Xinqiang Liang, Fayong Li

**Affiliations:** 1 College of Water Resources and Architectural Engineering, Tarim University, Alaer 843300, China; 120210084@taru.edu.cn (J.Z.); 120190106@taru.edu.cn (S.Z.);; 2 College of Architecture Engineering, Shandong Vocational and Technical University of Engineering, Jinan 250200, China; 3 School of Environmental Studies, China University of Geosciences, Wuhan 430074, China; huangwx@cug.edu.cn; 4 Department of Chemical & Materials Engineering, University of Auckland, Auckland 0926, New Zealand; 5 Key Laboratory of Environment Remediation and Ecological Health, Ministry of Education, College of Environmental and Resources Sciences, Zhejiang University, Hangzhou 310058, China

**Keywords:** cobalt ferrite, bismuth nitrate, photocatalysis, antibiotics, peroxymonosulfate, property

## Abstract

The widespread environmental contamination resulting from the misuse of tetracycline antibiotics (TCs) has garnered significant attention and study by scholars. Photocatalytic technology is one of the environmentally friendly advanced oxidation processes (AOPs) that can effectively solve the problem of residue of TCs in the water environment. This study involved the synthesis of the heterogeneous magnetic photocatalytic material of CoFe_2_O_4_/NaBiO_3_ via the solvothermal method, and it was characterized using different characterization techniques. Then, the photocatalytic system under visible light (Vis) was coupled with peroxymonosulfate (PMS) to explore the performance and mechanism of degradation of tetracycline hydrochloride (TCH) in the wastewater. The characterization results revealed that CoFe_2_O_4_/NaBiO_3_ effectively alleviated the agglomeration phenomenon of CoFe_2_O_4_ particles, increased the specific surface area, effectively narrowed the band gap, expanded the visible light absorption spectrum, and inhibited recombination of photogenerated electron–hole pairs. In the Vis+CoFe_2_O_4_/NaBiO_3_+PMS system, CoFe_2_O_4_/NaBiO_3_ effectively activated PMS to produce hydroxyl radicals (·OH) and sulfate radicals (SO_4_^−^). Under the conditions of a TCH concentration of 10 mg/L^−1^, a catalyst concentration of 1 g/L^−1^ and a PMS concentration of 100 mg/L^−1^, the degradation efficiency of TCH reached 94% after 100 min illumination. The degradation of TCH was enhanced with the increase in the CoFe_2_O_4_/NaBiO_3_ and PMS dosage. The solution pH and organic matter had a significant impact on TCH degradation. Notably, the TCH degradation efficiency decreased inversely with increasing values of these parameters. The quenching experiments indicated that the free radicals contributing to the Vis+CoFe_2_O_4_/NaBiO_3_+PMS system were ·OH followed by SO_4_^−^, hole (h^+^), and the superoxide radical (O_2_^−^). The main mechanism of PMS was based on the cycle of Co^3+^ and Co^2+^, as well as Fe^3+^ and Fe^2+^. The cyclic tests and characterization by XRD and FT-IR revealed that CoFe_2_O_4_/NaBiO_3_ had good degradation stability. The experimental findings can serve as a reference for the complete removal of antibiotics from wastewater.

## 1. Introduction

With the development of an intensive and large-scale breeding industry, the use of antibiotics is necessary to enhance farm productivity and the health of livestock and poultry, preventing diseases and promoting growth [1]. Tetracyclines (TCs) are broad-spectrum antibiotics widely adopted in livestock and poultry rearing due to their cost-effectiveness and benefits, such as the prevention and treatment of intestinal infections and growth promotion. However, the metabolic assimilation of TCs in animals is less than 30%, resulting in substantial excretion via feces and urine [2]. Consequently, substandard discharge of farm wastewater leads to the presence of TCs in aquatic environments. It has been reported that the residual TCs in the environment pose a significant threat to human health. Moreover, TCs can directly accumulate in the human body through the food chain, leading to endocrine disorders, joint diseases, central nervous system defects, and other diseases [3]. Furthermore, continuous environmental exposure to sub-therapeutic concentrations of TCs fosters selective pressure, facilitating the proliferation of antibiotic-resistant genes (ARGs) and endangering ecosystem stability and public health [4,5]. Current conventional physical removal techniques, such as adsorption and membrane filtration, are inadequate for the complete elimination of TCs, achieving merely a phase transfer rather than a true removal [6,7]. Similarly, traditional biological treatment technologies are also unable to effectively remove TCs, such as activated sludge, which mainly removes them by adsorption rather than biodegradation [8,9]. A study found that there are a large number of ARGs stored in the sludge produced by activated sludge and biofilm processes [10]. Therefore, it is urgent to find a method that can remove TCs from water quickly, safely, and completely.

In recent years, advanced oxidation processes (AOPs) based on free radicals with high redox potential have been recognized as an effective and promising method for removing environmental risks caused by antibiotics [11,12]. Among them, photocatalytic oxidation technology using abundant solar energy has attracted wide attention due to its great potential in solving the current environmental pollution problems as well as energy shortage [13]. Bismuth-based semiconductor photocatalytic materials such as bismuth molybdate (Bi_2_MoO_6_) [14], bismuth tungstate (BiVO_4_) [15], and sodium bismuthate (NaBiO_3_) [16] have become the research hotspots of photocatalysts due to their unique layered structures, suitable forbidden bandwidths, tunable morphology, high photonicity, and excellent electron transport efficiency [17]. Generally, Bi^5+^-containing oxides have a more vital ability to absorb visible light than Bi^3+^-containing oxides. NaBiO_3_ belongs to perovskite-type complex oxide (ABO_3_), which is one of the most common Bi^5+^-containing compounds [18] and has strong oxidation and photocatalytic abilities [19]. However, NaBiO_3_ photocatalysts have limitations such as high electron–hole pair recombination rate [20] and inability to be recycled, which limit their application in practice. Various strategies, including metal doping and the creation of heterojunctions, have been implemented to enhance the photocatalytic efficiency of NaBiO_3_. Wu [21] adeptly anchored nano-flower-like NaBiO_3_ onto g-C_3_N_4_ using a straightforward hydrothermal method, resulting in the formation of a direct Z-scheme g-C_3_N_4_/NaBiO_3_ heterojunction. This structure exhibited remarkable degradation capabilities for Tetracycline (TC) and notable stability. The internal electric field within the heterojunction promotes effective charge separation, while also providing a high redox potential for the photogenerated carriers. This facilitates the generation of additional reactive oxygen species (·O_2_^−^ and ·OH), leading to rapid TC degradation. A study found that the introduction of Fe^3+^ ions significantly reduces the recombination rate of photoinduced electron–hole pairs in NaBiO_3_, markedly improving its photocatalytic performance. Following 100 min of exposure, the photocatalytic degradation rate constant for TCH with Fe^3+^-doped NaBiO_3_ was found to be double that of the Vis+NaBiO_3_ system [22]. Wu [23] synthesized a NaBiO_3_/Poly (N-methylaniline) (PNMA) composite through a one-pot method, resulting in flake-like NaBiO_3_/PNMA photocatalysts. These composites, formed via in situ polymerization, exhibit strong interfacial effects that induce oxygen vacancy generation and an upward shift in the energy band structure. Consequently, these modifications significantly boost charge separation and light absorption efficiencies, showcasing superior catalytic activity in the oxidative degradation of TC. In order to solve the problem of the difficult recovery of NaBiO_3_, magnetic nanoparticles can be selected as carriers to obtain efficient and recyclable bismuth-based materials for water pollution treatment.

Cobalt ferrite (CoFe_2_O_4_), a typical spinel-type ferrite, has gained recognition as a potent catalyst for the degradation of organic pollutants in recent years. Its excellent catalytic efficiency is attributed to the synergistic effects of cobalt (Co) and iron (Fe), serving as active sites. Moreover, the integration with visible light irradiation enhances its catalytic capabilities [24]. Additionally, CoFe_2_O_4_ not only catalyzes peroxymonosulfate (PMS) synergistically under light irradiation, but also demonstrates outstanding electrocatalytic performance. This is characterized by high electronic conductivity, facilitation of charge carrier transfer, and reduction in electronic overpotential [25]. Most notably, CoFe_2_O_4_ features excellent magnetic recyclability [26], underscoring its potential for repeated use in catalytic processes. Utilizing CoFe_2_O_4_ in the synthesis of magnetic composite photocatalysts presents a commendable strategy. With a band gap ranging from 1 to 3 eV and inherent ferromagnetism, CoFe_2_O_4_ facilitates swift separation from the liquid–solid system under an external magnetic field. This property has led to its widespread application in photocatalytic oxidation technologies [27]. Al-Musawi [28] demonstrated that loading CoFe_2_O_4_ onto multi-walled carbon nanotubes reduced the band gap from 3.2 eV to 2.1 eV while simultaneously enhancing the light absorption rate. Lin [29] prepared CoFe_2_O_4_-modified BiOCl hierarchical microspheres, which, under mild light irradiation, exhibited piezoelectric photocatalytic reaction rates for TCH, bisphenol A, and phenol significantly higher than those observed in standard photocatalysis. Jing [30] developed a visible-light-driven magnetic CoFe_2_O_4_/Ag/Ag_3_VO_4_ photocatalyst via a hydrothermal method, which effectively degraded methyl orange and TC and eradicated Escherichia coli, maintaining stable degradation ability and crystal structure. A highly efficient S-scheme heterojunction CoFe_2_O_4_/NiFe_2_O_4_ was developed by integrating CoFe_2_O_4_ and NiFe_2_O_4_ nanosheets [31]. The 5%-CoFe_2_O_4_/NiFe_2_O_4_ demonstrated remarkable photocatalytic degradation capabilities for TC, achieving a removal rate of 76.1% within 60 min. This enhancement in photocatalytic performance is primarily attributed to the formation of the S-scheme heterojunction, which significantly increases the capacity for visible light absorption and facilitates charge separation. Gogoi [32] reported that a catalyst composed of CoFe_2_O_4_ nanoparticles and g-C_3_N_4_ nanosheets (CF-gC_3_N_4_) displayed exceptional catalytic efficiency in degrading various widely used antibiotics. The catalyst achieved complete degradation of TCH under simulated sunlight irradiation within 12 min, and under microwave irradiation, the degradation was accomplished in just 1 min. The catalyst can be easily separated using magnetic methods, exhibits a catalytic efficiency of up to 91%, and maintains good stability. Furthermore, CoFe_2_O_4_ can has been successfully combined with various materials like TiO_2_ [33], ZnO [34], graphene [35], g-C_3_N_4_ [36], and BiVO_4_ [37], enhancing photocatalytic activity to varying degrees. Specifically, CoFe_2_O_4_ nanoparticles on loaded NaBiO_3_ surfaces not only boost the photocatalytic performance of composite catalysts, but also contribute to preventing the direct or indirect emission of pollutants, thereby mitigating primary and secondary environmental pollution.

Achieving efficient degradation with a singular photocatalytic technology proves challenging. However, merging two distinct AOPs circumvents their individual limitations while leveraging their synergistic effect to further augment pollutant removal efficiency. SO_4_^−^·-based persulfate AOPs (SR-AOPs) have recently emerged as a focal point in organic wastewater treatment in recent years, offering advantages over ·OH and SO_4_^−^·, including a higher redox potential (2.5–3.1 V) [38], longer half-life (30–40 μs) [39], broader pH stability, and enhanced robustness. CoFe_2_O_4_ has been widely used to activate persulfate (PS) for catalytic oxidation degradation of various pollutants [40,41]. Moreover, visible light activation of PS presents a quicker, more reliable method. For instance, Li [40] used nano-CoFe_2_O_4_-activated PMS to degrade atrazine (ATZ), achieving over 99% removal within 30 min. Song [41] explored the Triphenyl phosphate (TPhP) degradation via CoFe_2_O_4_-activated PMS, demonstrating a 99.5% removal efficiency under ambient conditions, underscoring the efficacy of the CoFe_2_O_4_ -activated PMS process for TPhP degradation. Thus, the photocatalytic technology coupled with persulfate technology using CoFe_2_O_4_/NaBiO_3_ as the catalyst can significantly enhance removal efficiency and reaction speed.

This study introduces CoFe_2_O_4_/NaBiO_3_ heterogeneous magnetic photocatalysts, synthesized via the solvothermal method. CoFe_2_O_4_, NaBiO_3_, and CoFe_2_O_4_/NaBiO_3_ were characterized by various analytical techniques. These catalysts, coupled with PMS, formed Vis+CoFe_2_O_4_/NaBiO_3_+PMS systems, providing a synergistic reaction pathway for TCH removal. The effects of reaction conditions and fulvic acid (FA) coexistence on TCH degradation were investigated, and the main free radical species promoting TCH degradation in a collaborative system were determined. Subsequent cyclic experiments evaluated the composite photocatalyst’s stability and reusability. The magnetic nature of CoFe_2_O_4_/NaBiO_3_ significantly eases catalyst recycling challenges, paving the way for future research on the development of bismuth-based materials with excellent performance and novel solutions for persistent organic pollution eradication.

## 2. Results and Discussion

### 2.1. Characterization of Photocatalysts

**Figure 1:**
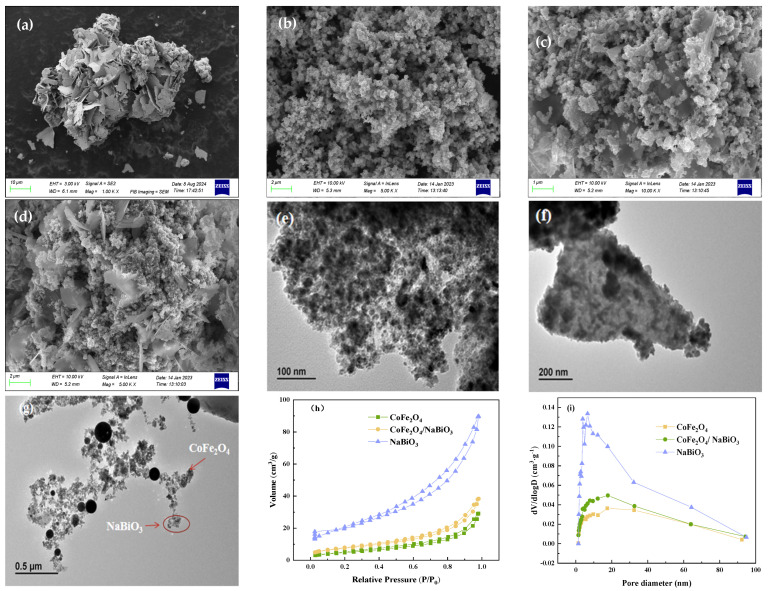
SEM and TEM images, adsorption–desorption curves, and pore size distributions of CoFe_2_O_4_, NaBiO_3_, and CoFe_2_O_4_/NaBiO_3_. (**a**) NaBiO_3_ at 10 μm; (**b**) CoFe_2_O_4_ at 2 μm; (**c**,**d**) CoFe_2_O_4_/NaBiO_3_ at 1 μm and 2 μm, respectively. TEM images of samples: (**e**) CoFe_2_O_4_; (**f**) NaBiO_3_; (**g**) CoFe_2_O_4_/NaBiO_3_. (**h**) Adsorption–desorption curve of samples. (**i**) Pore size distribution of samples.

Scanning electron microscopy (SEM) images of CoFe_2_O_4_, NaBiO_3_, and CoFe_2_O_4_/NaBiO_3_ are presented in Figure 1a–d, elucidating the morphological characteristics of the synthesized samples. The commercially available NaBiO_3_ (Figure 1a) exhibits a layered structure composed of nanosheets varying in shape and size. This expansive and smooth surface area of NaBiO_3_ provides an optimal substrate for forming composite with other materials. The CoFe_2_O_4_ surface is characterized by uniformly shaped spherical nanoparticles, albeit with some degree of agglomeration, which may obscure active sites and consequently dampen its catalytic efficacy (Figure 1b). This agglomeration is postulated to stem from the inherent strong magnetic properties of CoFe_2_O_4_. A comparative analysis of Figure 1a–d reveals the successful deposition of CoFe_2_O_4_ nanoparticles onto layered NaBiO_3_ flakes. This arrangement allows NaBiO_3_ to effectively disperse the CoFe_2_O_4_ particles, mitigating their natural tendency to agglomerate, thereby unveiling active sites on CoFe_2_O_4_/NaBiO_3_ and enhancing its photocatalytic efficiency.

To further corroborate the successful integration of CoFe_2_O_4_ nanoparticles with NaBiO_3_, transmission electron microscope (TEM) images of CoFe_2_O_4_, NaBiO_3_, and CoFe_2_O_4_/NaBiO_3_ are provided in Figure 1e–g. The TEM image of pure CoFe_2_O_4_ (Figure 1e) reveals relatively uniform nanoparticles with an average size of about 20 nm, prone to aggregation due to magnetic properties. The NaBiO_3_ (Figure 1f) is predominantly sheet-like, with a thickness ranging from 10 to 15 nm and a length from 200 to 600 nm. Its overall structure is thin, featuring uneven thickness and a coarse surface, conducive to CoFe_2_O_4_ loading. The TEM image of the CoFe_2_O_4_/NaBiO_3_ composite (Figure 1g) distinctly shows the NaBiO_3_ surface adorned with small CoFe_2_O_4_ nanoparticles, highlighting a unique microstructure that optimizes the interface between NaBiO_3_ and CoFe_2_O_4_. This configuration significantly improves the separation efficiency of photogenerated electrons and holes, thereby augmenting the photocatalytic performance of the composite [30].

Brunauer–Emmett–Teller (BET) analyses were conducted to determine the specific surface area and pore size distribution of CoFe_2_O_4_, NaBiO_3_, and CoFe_2_O_4_/NaBiO_3_. As shown in Figure 1h, all catalysts exhibited type IV isotherms with H3 hysteresis loops, indicative of mesoporous structures and a high adsorption capacity at elevated P/P0 ratios, likely due to slit pores formed by particle aggregation. Among them, NaBiO_3_ exhibited the highest adsorption capacity, succeeded by CoFe_2_O_4_/NaBiO_3_, with CoFe_2_O_4_ showing the least. This suggests that the presence of CoFe_2_O_4_ incorporation obstructs numerous pores within NaBiO_3_. The specific surface areas for NaBiO_3_, CoFe_2_O_4_, and CoFe_2_O_4_/NaBiO_3_ were measured at 72.14 m^2^·g^−1^, 17.38 m^2^·g^−1^, and 26.94 m^2^·g^−1^ respectively, indicating that NaBiO_3_ serves as an effective medium for dispersing CoFe_2_O_4_ particles and amplifying the specific surface area to expose a greater number of active sites. As shown in Figure 1i, the pore size distribution of NaBiO_3_ predominantly ranges between 5 and 10 nm, whereas for CoFe_2_O_4_ and CoFe_2_O_4_/NaBiO_3_, it is chiefly between 10 and 30 nm.

**Figure 2:**
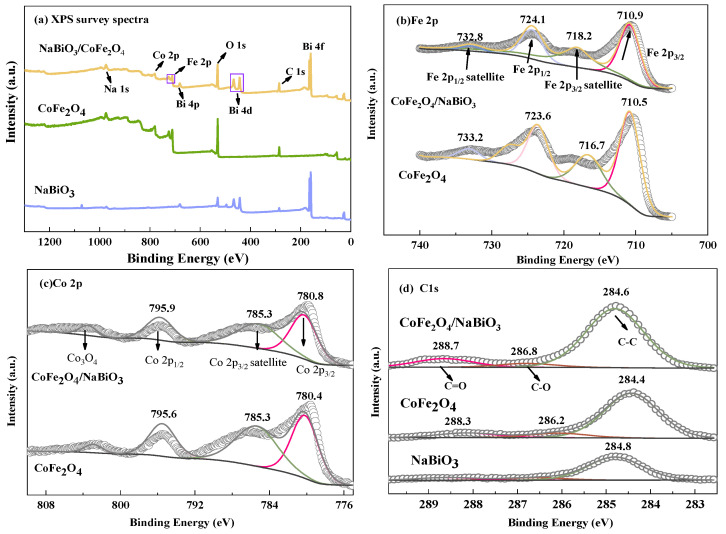

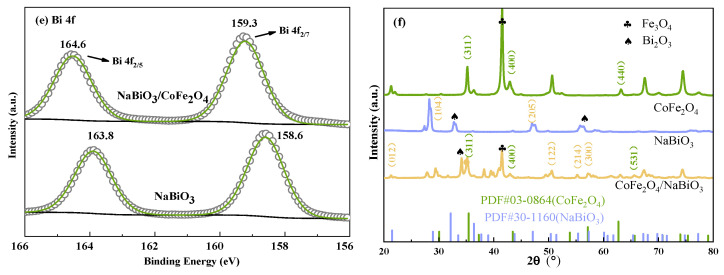
XPS spectra and XRD patterns of CoFe_2_O_4_, NaBiO_3_, and CoFe_2_O_4_/NaBiO_3_. (**a**) XPS survey spectra; (**b**) Fe 2p orbital diagram; (**c**) Co 2p orbital diagram; (**d**) C 1s orbital diagram; (**e**) Bi 4f orbital diagram; (**f**) XRD pattern.

Figure 2 shows the X-ray photoelectron spectroscopy (XPS) spectra for CoFe_2_O_4_, NaBiO_3_, and CoFe_2_O_4_/NaBiO_3_ composite. Analysis of Figure 2a reveals the detection of elements such as Co, Fe, C, O, and Bi within the CoFe_2_O_4_/NaBiO_3_ composite. Figure 2b–e display high-resolution XPS spectra for the elements Co, Fe, C, and Bi, respectively. The high-resolution XPS spectra of the CoFe_2_O_4_ (Figure 2b) identifies four prominent peaks corresponding to Fe 2p, with the peaks at 710.5 eV (and a satellite peak at 716.7 eV) and 723.6 eV (with a satellite peak at 733.2 eV) attributed to Fe 2p_3/2_ and Fe 2p_1/2_, respectively. The peak at 710.5 eV indicates the presence of Fe^2+^, and the binding energy of 723.6 eV aligns with the characteristic value of Fe^3+^ [42]. Upon forming the CoFe_2_O_4_ and NaBiO_3_ composite, these characteristic peaks shift to 710.9, 718.2, 724.1, and 732.8 eV, respectively, indicating a transformation in the chemical environment of CoFe_2_O_4_/NaBiO_3_ and confirming a strong interaction between CoFe_2_O_4_ and NaBiO_3_. Figure 2c illustrates three distinct peaks of Co 2p, centered at 780.4 eV (with a satellite peak at 785.3 eV) and 795.6 eV, corresponding to the orbital peaks of Co 2p_3/2_ and Co 2p_1/2_, respectively, primarily associated with Co^2+^ [43]. The slight shift in these orbital peaks towards higher binding energies in the composite suggests a decrease in electron density, indicative of a robust integration of the elements. The detection of Co 2p and Fe 2p peaks confirms the presence of spinel CoFe_2_O_4_ within the composite [44]. The C1s XPS spectra for both CoFe_2_O_4_ and CoFe_2_O_4_/NaBiO_3_ are differentiated into three peaks of 284.6 eV, 286.8 eV, and 288.7 eV (Figure 2d), corresponding to C-C, C=O and C-O, respectively [45]. In Figure 2e, the Bi element is precisely identified, with Bi 4f exhibiting two peaks at binding energies of 158.6 and 163.8 eV, characteristic of Bi 4f_7/2_ and Bi 4f_5/2_, respectively, and indicative of Bi^5+^ [46]. For the composite, these peaks shift to 159.3 and 164.6 eV, respectively, aligning with the properties of Bi^3+^ [47], thereby evidencing a valence state change of the Bi element on the material’s surface and confirming the presence of Bi_2_O_3_ as detected in XRD analyses. This shift and the elemental migration observed in the composite compared to the pure substances suggest that CoFe_2_O_4_ and NaBiO_3_ form a heterojunction upon combination, facilitating the transfer of photogenerated electrons.

To further elucidate the phase and crystal structure of CoFe_2_O_4_, NaBiO_3_, and CoFe_2_O_4_/NaBiO_3_, XRD analysis was conducted (Figure 2f). For CoFe_2_O_4_ nanoparticles, peaks were observed at 2θ values of 35.451°, 43.472°, and 62.726°, corresponding to the (311), (400), and (440) crystal planes of spinel CoFe_2_O_4_ (PDF#03-0864) alongside the detected Fe_3_O_4_ peaks. For pure NaBiO_3_ nanosheets, characteristic peaks were observed at 2θ values of 28.927° and 47.123°, consistent with the crystal planes (104) and (205) of NaBiO_3_ (PDF#30–1160), with Bi_2_O_3_ peaks also identified. Within the CoFe_2_O_4_/NaBiO_3_ composite catalyst, NaBiO_3_ characteristic peaks were observed at 2θ values of 21.498°, 51.407°, 55.367°, and 57.322°, corresponding to (012), (122), (214), and (300), respectively. Additionally, CoFe_2_O_4_ peaks corresponding to the (311), (400), and (531) crystal faces were observed at 2θ values of 34.451°, 43.472°, and 65.701°, respectively. Fe_3_O_4_ and Bi_2_O_3_ were also observed in the composite. XRD results indicate that the magnetic photocatalytic material CoFe_2_O_4_/NaBiO_3_ was successfully prepared.

**Figure 3:**
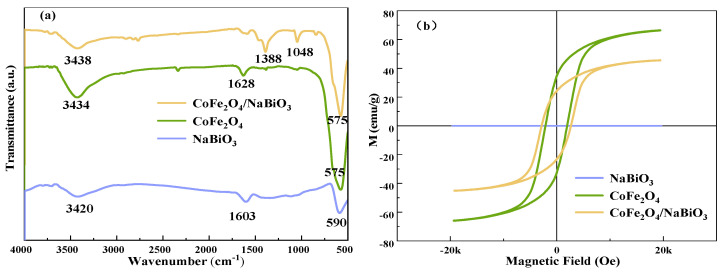
FT-IR and VSM spectra of CoFe_2_O_4_, NaBiO_3_, and CoFe_2_O_4_/NaBiO_3_. (**a**) FT-IR spectra; (**b**) VSM spectra.

The Fourier transform infrared spectroscopy (FT-IR) spectra of CoFe_2_O_4_, NaBiO_3_, and CoFe_2_O_4_/NaBiO_3_ are depicted in Figure 3a. The spectra reveal a broad absorption peak between 3300 and 4450 cm^−1^ alongside absorption peaks within the 1400–1649 cm^−1^ range, likely attributable to the stretching vibration of the O-H group in water molecules adsorbed on the catalyst surface [48,49]. For NaBiO_3_, an absorption peak at 590 cm^−1^ is mainly due to the vibration of Bi-O bonds [50]. An observable peak at 1048 cm^−1^ might represent the stretching vibration of C-O in the metal–organic chelate, resulting from the chelation of ethylene glycol and metal ions during the synthesis of CoFe_2_O_4_. The peak range of 1380−1390 cm^−1^ could be associated with the formation of-C-O-H in the in-plane band, a result of the interaction between CoFe_2_O_4_ and NaBiO_3_. Additionally, the absorption peak at 575 cm^−1^ is likely indicative of Fe-O and Co-O bond vibrations within CoFe_2_O_4_. The comparative analysis of peaks between CoFe_2_O_4_/NaBiO_3_ and CoFe_2_O_4_ further confirms the successful synthesis of the CoFe_2_O_4_/NaBiO_3_ composite.

The vibrating sample magnetometer (VSM) profiles for NaBiO_3_, CoFe_2_O_4_, and CoFe_2_O_4_/NaBiO_3_, presented in Figure 3b, demonstrate that NaBiO_3_ exhibits negligible saturation magnetization strength, in contrast to CoFe_2_O_4_ and CoFe_2_O_4_/NaBiO_3_, which display pronounced hysteresis line trends. At a magnetic field strength of 20 k, the saturation magnetization reaches 66.4 emu·g^−1^ for CoFe_2_O_4_ and 45.5 emu·g^−1^ for CoFe_2_O_4_/NaBiO_3_. The higher saturation magnetization and coercivity of CoFe_2_O_4_ compared to CoFe_2_O_4_/NaBiO_3_ indicates robust magnetism in both catalysts. However, the magnetism of CoFe_2_O_4_/NaBiO_3_ is slightly reduced upon the incorporation of NaBiO_3_, a factor that does not impede the magnetic recovery of the composite photocatalysts.

**Figure 4:**
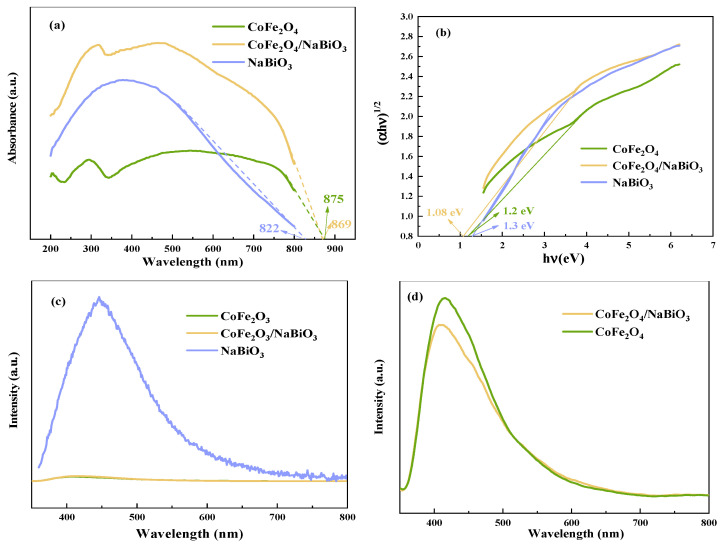
UV-Vis DRS and PL spectra of CoFe_2_O_4_, NaBiO_3_, and CoFe_2_O_4_/NaBiO_3_. (**a**) UV-Vis DRS spectra; (**b**) the forbidden bandwidth diagram; (**c**) PL spectra; (**d**) local amplification of PL spectra.

The ultraviolet-visible diffuse reflectance spectroscopy (UV-Vis DRS) and photoluminescence spectroscopy (PL) spectra of CoFe_2_O_4_, NaBiO_3_, and CoFe_2_O_4_/NaBiO_3_ are showcased in Figure 4. Analysis of Figure 4a reveals that both CoFe_2_O_4_, NaBiO_3_, and CoFe_2_O_4_/NaBiO_3_ are capable of both ultraviolet and visible light, with CoFe_2_O_4_/NaBiO_3_ exhibiting enhanced absorption intensity, particularly favorable for visible light absorption. The threshold wavelengths for CoFe_2_O_4_, NaBiO_3_, and CoFe_2_O_4_/NaBiO_3_ are 822, 875, and 869 nm, respectively, indicating absorption in the infrared spectrum for all mentioned catalysts. The bandwidths of CoFe_2_O_4_ and CoFe_2_O_4_/NaBiO_3_ were evaluated according to the Kubelka–Munk formula [51]: αhυ = A (hυ − Eg)^n/2^, where α is the absorption coefficient; h is Planck constant; υ is the frequency of incident light; and A is a constant. When the semiconductor is a direct transition semiconductor, n is 1; when the semiconductor is an indirect transition semiconductor, n is 4. The calculation results are shown in Figure 4b. The bandwidths of CoFe_2_O_4_, NaBiO_3_, and CoFe_2_O_4_/NaBiO_3_ are 1.1, 1.3, and 1.08 eV, respectively, proving that the combination of CoFe_2_O_4_ and NaBiO_3_ semiconductors can effectively reduce the forbidden bandwidth and, at the same time, enhance the absorption of visible light, thus improving the degradation performance of CoFe_2_O_4_/NaBiO_3_. Figure 4c shows the PL spectra of CoFe_2_O_4_, NaBiO_3_, and CoFe_2_O_4_/NaBiO_3_, reflecting the dynamics of photogenerated electron–hole pair separation and recombination at an excitation wavelength of 340 nm. NaBiO_3_ exhibits a strong emission peak at 450 nm, whereas CoFe_2_O_4_ and CoFe_2_O_4_/NaBiO_3_ show significant emission peaks near 410 nm. Notably, CoFe_2_O_4_/NaBiO_3_ substantially diminishes the intensity of the emission band, suggesting that the integration of CoFe_2_O_4_ and NaBiO_3_ effectively hinders the rapid recombination of electron–hole pairs, thereby augmenting the photocatalytic performance.

### 2.2. Properties of Photocatalytic Degradation of TCH in Different Systems

**Figure 5:**
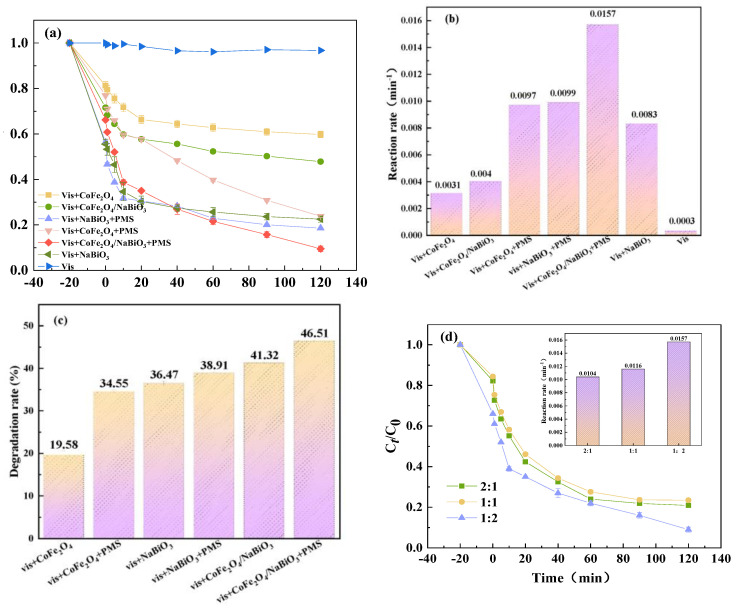
The degradation efficiency on TCH and the corresponding degradation rate constants. (**a**,**b**) The removal efficiency and reaction rate of different reaction systems; (**c**) degradation rates of TOC in different systems; (**d**,**e**) effects of different CoFe_2_O_4_ and NaBiO_3_ composite ratios on TCH removal efficiency and reaction rate. Conditions: [pH] unregulated, [TCH] = 10 mg/L^−1^, [PMS] = 100 mg/L^−1^, [catalyst] = 0.5 g/L^−1^.

To assess the photocatalytic performance of CoFe_2_O_4_, NaBiO_3_ and CoFe_2_O_4_/NaBiO_3_, various reaction systems were established for conducting TCH photocatalytic degradation experiments. The outcomes of these experiments are illustrated in Figure 5. Initially, an adsorption experiment under dark conditions was conducted with a TCH concentration of 10 mg/L^−1^. After 20 min, CoFe_2_O_4_ exhibited a TCH removal rate of merely 18.7%, whereas NaBiO_3_ achieved a removal efficiency of up to 45%. The CoFe_2_O_4_/NaBiO_3_ composite demonstrated a removal efficiency of 28.4%, indicating enhanced adsorption capacity compared to CoFe_2_O_4_ alone. This improvement is likely attributable to an increased specific surface area. However, the introduction of CoFe_2_O_4_ resulted in a slight reduction in adsorption performance compared to NaBiO_3_ alone, possibly due to CoFe_2_O_4_ occupying some of the narrow channels within the NaBiO_3_ nanosheets, thereby impacting its adsorption capabilities. Therefore, it can be inferred that it is difficult to achieve rapid and complete removal of TCH through the adsorption reaction relying solely on CoFe_2_O_4_, NaBiO_3_, and CoFe_2_O_4_/NaBiO_3_. Subsequent to the adsorption phase, a 500 W xenon lamp was activated to initiate the photocatalytic oxidation process. The results revealed that, after 120 min of illumination, TCH removal rates under single illumination were less than 5%. In contrast, the degradation efficiency for the Vis+CoFe_2_O_4_, the Vis+NaBiO_3_, and the Vis+CoFe_2_O_4_/NaBiO_3_ system reached 40.2%, 75%, and 52.2%, respectively. This indicates that CoFe_2_O_4_ possesses inherent photocatalytic activity, as evidenced by a 22.5% increase in TCH removal rate compared to adsorption alone. Moreover, the Vis+CoFe_2_O_4_/NaBiO_3_ system exhibited an enhanced photocatalytic performance compared to CoFe_2_O_4_ alone. A pseudo-first-order kinetic model was employed to calculate the photodegradation reaction kinetics, employing the formula ln (*C*_0_/*C_t_*) = *kt*, where *C*_0_ and *C_t_* represent the initial and time-dependent TCH concentration, respectively, and *k* is the kinetic constant [52]. The reaction rate for the Vis+CoFe_2_O_4_/NaBiO_3_ system increased from 0.0031 min^−1^ to 0.004 min^−1^, aligning with the observed efficiency improvements. The improvement of degradation efficiency and rate are attributed to increased effective light absorption and the reduced recombination of photobiogenic carriers.

Introducing PMS as an electron acceptor to the photocatalytic system was expected to further improve the degradation efficiency. After incorporating PMS, the TCH removal efficiency in the Vis+CoFe_2_O_4_ system surged by approximately 36%, reaching 76.2%, with the reaction rate escalating from 0.0031 min^−1^ to 0.0097 min^−1^. This indicates that the visible light in conjunction with CoFe_2_O_4_ effectively activate PMS, generating highly oxidation species and enhancing TCH degradation. In the Vis+CoFe_2_O_4_NaBiO_3_+PMS system, TCH removal soared to 61% after only 10 min of illumination and reached 90.5% after 2 h, marking a 38% increase in final degradation efficiency compared to the Vis+CoFe_2_O_4_/NaBiO_3_ system without PMS. The reaction rate also increased from 0.004 min^−1^ to 0.0157 min^−1^, demonstrating significant improvements in the synergistic interaction between photocatalysis and PMS activation. This synergy is primarily due to PMS’s ability to capture photoinduced electrons and mitigate the recombination of photocatalyst carriers, thereby facilitating the generation of reactive oxygen species (ROS) and enhancing TCH degradation [53].

Additionally, the mineralization rate of organic matter is a critical metric for evaluating catalytic performance. The total organic carbon (TOC) analyzer (TOC, enviro TOC, Elementar, Germany) was employed to analyze the mineralization degree of the aforementioned systems on TCH (Figure 5c). This finding is in congruence with Figure 5a. The outcomes manifested that, subsequent to 120 min of illumination, the TOC removal rate of the Vis+CoFe_2_O_4_ system was 19.58%, and the rate of the Vis+CoFe_2_O_4_/NaBiO_3_ system incrementally escalated to 27.15%, yet still remained inferior to that of the Vis+NaBiO_3_ system (36.47%). This could potentially be attributed to the introduction of CoFe_2_O_4_, which diminishes the specific surface area of NaBiO_3_, thereby impinging upon its photocatalytic performance. In contrast to the solitary photocatalytic oxidation systems, the photocatalytically coupled PMS exhibits a higher TOC removal rate, particularly in the Vis+CoFe_2_O_4_/NaBiO_3_+PMS system (46.51%), signifying that upon the addition of PMS, more active substances for degrading TOC are engendered within the system, giving rise to a higher removal rate. This further substantiates the preponderance of photocatalytically coupled PMS systems in TCH degradation and mineralization.

The catalytic efficiency of CoFe_2_O_4_/NaBiO_3_ composite catalysts across various ratios was evaluated to identify the formulation with the highest efficacy. The results are depicted in Figure 5d. Analysis of the figure reveals that the catalytic system comprising Vis+CoFe_2_O_4_/NaBiO_3_+PMS demonstrated the lowest TCH removal rate of 76.8% when the NaBiO_3_ to CoFe_2_O_4_ ratio is 1:1. Conversely, when the ratio was adjusted to 2:1, the degradation rate increased to 90.5% after 120 min, with the corresponding quasi-first-order kinetic rate constant k reaching a peak value of 0.0157 min^−1^. These findings suggest that enhancing the CoFe_2_O_4_ content within the composite catalyst leads to improved catalytic performance. This improvement can be attributed to the increase in active sites resulting from a higher CoFe_2_O_4_ loading ratio, which facilitates the photocatalytic activation of PMS, generating a greater number of active species. Furthermore, NaBiO_3_ aids in the transfer process of carriers to the surface, culminating in the most effective TCH removal rate.

**Figure 6:**
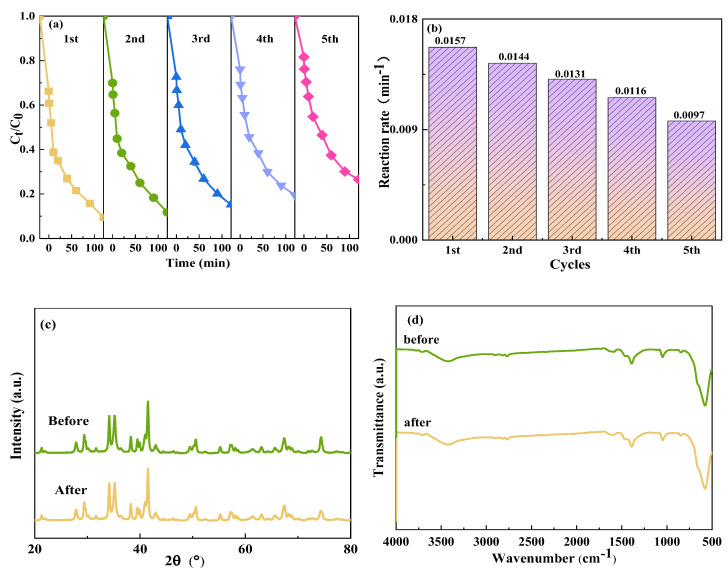
Stability of CoFe_2_O_4_/NaBiO_3_ and XRD and FT-IR spectra before and after photocatalytic reaction. (**a**,**b**) The TCH degradation efficiency and rate in the cyclic experiments; (**c**,**d**) XRD and FT-IR spectra before and after cycling. Conditions: [pH] unregulated, [TCH] = 10 mg/L^−1^, [PMS] = 100 mg/L^−1^, [catalyst] = 0.5 g/L^−1^.

In practical applications, the effectiveness of photocatalysts significantly depends on their stability. Cyclic experiments were carried out to study the stability of CoFe_2_O_4_/NaBiO_3_. Leveraging the magnetic properties of the photocatalyst facilitated its easy recycling. It was found that the CoFe_2_O_4_/NaBiO_3_ composite exhibited a recovery rate of 90.3%, effectively addressing the challenge of recovering NaBiO_3_ due to its strong magnetic properties. The durability of the recovered CoFe_2_O_4_/NaBiO_3_ was assessed through degradation tests conducted over five cycles, with the results depicted in Figure 6a,b. After five cycles, the degradation efficiency of TCH in the reaction system of Vis+CoFe_2_O_4_/NaBiO_3_+PMS decreased from 90.5% to 73.5%, and the reaction rate decreased from 0.0157 min^−1^ to 0.0097 min^−1^. Concurrently, the adsorption removal rate of CoFe_2_O_4_/NaBiO_3_ for TCH experienced a decline of approximately 13% after five cycles (Figure 6a). This suggests that multiple cycles significantly undermined the adsorption performance of the composite catalyst. The presence of more macropores in CoFe_2_O_4_/NaBiO_3_ was beneficial for the entry and adsorption of TCH molecules at active site. However, upon light activation, the active species produced through photocatalysis and PMS activation encountered challenges in accessing the catalyst’s interior, leading to the occupation of the active site and a subsequent reduction in adsorption performance. Consequently, both the adsorption efficiency and the catalytic performance of CoFe_2_O_4_/NaBiO_3_ witnessed a decline, which can be attributed to losses incurred during the catalyst’s magnetic recovery process and the adsorption of intermediate degradation products on the catalyst surface. These factors contribute to a reduction in the catalyst’s specific surface area and reactive active sites, ultimately hindering the degradation of organic matter [54]. XRD and FT-IR analyses were carried out on CoFe_2_O_4_/NaBiO_3_ before and after usage, and the results are shown in Figure 6c,d. The analyses revealed no emergence of new characteristic peaks following photocatalysis, with only a slight reduction in existing peaks observed. Therefore, it can be concluded that the synthesized CoFe_2_O_4_/NaBiO_3_ is a reusable and stable high-performance photocatalyst under visible light.

### 2.3. Effect of Experimental Conditions on the Degradation of TCH in the Reaction System

#### 2.3.1. Effect of Catalyst and Oxidant Concentration

**Figure 7:**
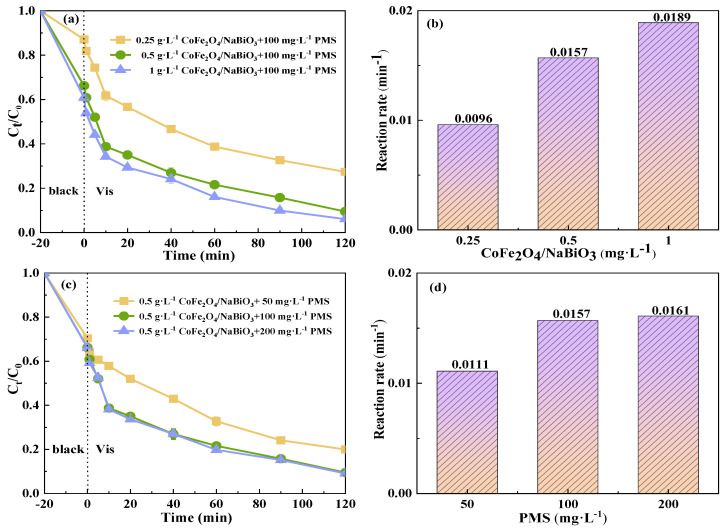
The effects of CoFe_2_O_4_/NaBiO_3_ and PMS concentrations on the degradation of TCH in the Vis+CoFe_2_O_4_/NaBiO_3_+PMS system. (**a**,**b**) The effects of CoFe_2_O_4_/NaBiO_3_ concentration on TCH removal efficiency and rate. Conditions: [pH] unregulated, [TCH] = 10 mg/L^−1^, [PMS] = 100 mg/L^−1^, [catalyst] = 0.25, 0.5 and 1.0 g/L^−1^. (**c**,**d**) The effects of PMS concentration on TCH removal efficiency and rate. Conditions: [pH] unregulated, [TCH] = 10 mg/L^−1^, [catalyst] = 0.5 g/L^−1^, [PMS] = 50, 100, 200 mg/L^−1^.

The degradation efficiency of TCH in the Vis+CoFe_2_O_4_/NaBiO_3_+PMS system increased with the increase in the catalyst concentration (Figure 7). When the added concentration of CoFe_2_O_4_/NaBiO_3_ was increased from 0.25 g·L^−1^ to 0.5 g·L^−1^, the adsorption removal efficiency of TCH increased by about 20% after 20 min of dark reaction. After photocatalytic degradation, the degradation efficiency greatly improved from 72.6% to 90.5%, and the reaction rate improved from 0.0096 min^−1^ to 0.0157 min^−1^. When the catalyst addition concentration was increased from 0.5 g·L^−1^ to 1 g·L^−1^, the degradation efficiency was only increased from 90.5% to 94%, and the reaction rate from 0.0157 min^−1^ to 0.0189 min^−1^. Although the degradation efficiency was improved, the increase was slight. The possible reason is that when the concentration of CoFe_2_O_4_ is higher, although it can provide more active sites, it also increases the turbidity of the reaction solution to a certain extent, which affects the reaction of CoFe_2_O_4_/NaBiO_3_ with light. Moreover, CoFe_2_O_4_ formed Fe^2+^ during the activated PMS process and reacted with the generated SO_4_−· and ·OH to consume the active species. Therefore, when the dosage of CoFe_2_O_4_ increases, more Fe^2+^ is produced. With a fixed dosage of PMS, the generated Fe^2+^ consumes more free radicals, which in turn affects the removal of TCH.

In the Vis+CoFe_2_O_4_/NaBiO_3_+PMS system, as the source of SO_4_−·, the concentration of PMS will directly affect the degradation efficiency of TCH. As depicted in Figure 7c,d, with the concentration of PMS in the system increasing, the degradation efficiency and reaction rate of TCH were correspondingly enhanced. Additionally, prior research has similarly documented a positive correlation between the degradation rate of pollutants and the concentration of PS [55]. When the PMS concentration was 50 mg/L^−1^, the TCH degradation efficiency and reaction rate after 120 min of light exposure were 80% and 0.0111 min^−1^, respectively. With the concentration rising to 100 mg/L^−1^, both the TCH degradation efficiency and reaction rate significantly increased, reaching 90.5% and 0.0157 min^−1^. The observed increase in TCH degradation rate may be attributed to the activation of more sulfate ions through the addition of additional PMS, leading to the formation of SO_4_^−^·(Equation (1)). However, as the concentration of PMS increased to 200 mg/L^−1^, the observed increase in both the TCH degradation rate and the reaction rate diminished, with values of 91% and 0.0161 min^−1^, respectively, which could be attributed to the fact that the concentration of PMS was too high when the concentration of PMS was added to 200 mg/L^−1^. Large amounts of SO_4_^−^·and OH produced by PMS were quenched with the excess PMS within a short time to generate SO^5−^·(Equations (2) and (3)), which had weakened oxidation capacity, resulting in a minor increase in TCH degradation efficiency and rate and a lower SO_4_^−^ utilization rate [56].

2HSO_5_^−^ + e^+^ → SO_4_^−^·+ SO_4_^2−^ + H^+^ (1)

SO_4_^−^·+ HSO_5_^−^ → SO_5_^−^·+SO_4_^2−^ + H^+^ (2)

OH + HSO_5_^−^ → SO_5_^−^·+ H_2_O (3)

#### 2.3.2. Effect of TCH Concentration

**Figure 8:**
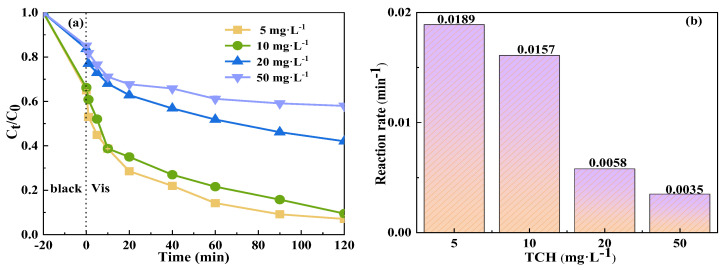
Effect of initial TCH concentration on the degradation of TCH in the Vis+CoFe_2_O_4_/NaBiO_3_+PMS system. (**a**) Degradation efficiency; (**b**) reaction rate. Conditions: [pH] unregulated, [TCH] = 5, 10, 20, 50 mg/L^−1^, [PMS] = 100 mg/L^−1^, [catalyst] = 0.5 g/L^−1^.

Figure 8 shows the effect of the initial TCH concentration on its degradation by the Vis+CoFe_2_O_4_/NaBiO_3_+PMS system. As the initial concentration of TCH was elevated from 5 mg/L^−1^ to 50 mg/L^−1^, the degradation efficiency of TCH by the system declined from 93% to 42%, representing a decrease of approximately 49%. Concurrently, the reaction rate decreased from 0.0189 min^−1^ to 0.0035 min^−1^. This decrease can be attributed to the high initial concentration of pollutants, which likely hampered the entry of photons into the photocatalytic reaction as well as the generation of reactive radicals [57,58,59]. In addition, given that the production of active species by CoFe_2_O_4_/NaBiO_3_ remained constant at specific concentrations, an increase in TCH resulted in a corresponding rise in degradation intermediates. The intermediates compete with TCH molecules, further impeding the reaction rate.

#### 2.3.3. Effect of Initial pH

**Figure 9:**
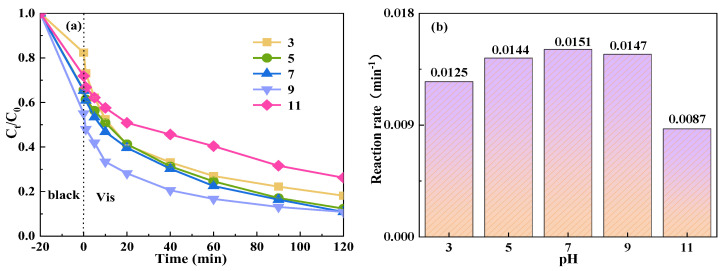
Effect of initial pH value on the degradation of TCH in the Vis+CoFe_2_O_4_/NaBiO_3_+PMS system. (**a**) The TCH degradation efficiency diagram. (**b**) The reaction rate diagram. Conditions: [pH] = 3, 5, 7, 9, 11; [TCH] = 10 mg/L^−1^, [PMS] = 100 mg/L^−1^, [catalyst] = 0.5 g/L^−1^.

The removal efficiency of TCH showed a trend of initial increase followed by a subsequent decrease with varying pH levels, as shown in Figure 9. Specifically, as the pH value increased from 3 to 7, the degradation efficiency of TCH improved from 81.9% to 89.2%, and the reaction rate also rose from 0.0125 min^−1^ to 0.0151 min^−1^. However, upon increasing the pH from 7 to 9, the degradation efficiency remained constant at 89.2%, yet the reaction rate slightly decreased to 0.0147 min^−1^. Further elevation of the pH from 9 to 11, which represents an extremely alkaline condition, resulted in a significant reduction in degradation efficiency to 73.8%, with the reaction rate also falling to 0.0087 min^−1^. These observations indicate that the degradation efficiency and rate are optimal under neutral pH conditions. In contrast, under extremely acidic conditions, the performance is superior to that under alkaline conditions. This disparity is due to the reaction of OH^−^ with SO_4_^−^· in alkaline solutions to produce ·OH, which becomes the predominant active species [60]. The redox potential of ·OH (1.8–2.7 V) was significantly lower than that of SO_4_^−^·(2.5–3.1 V), implying a reduced oxidation ability of OH [61] and, consequently, a diminished degradation efficiency of TCH. Conversely, under acidic conditions, PMS mainly exists in the form of H_2_SO_5_, and the catalyst is less effective in activating PMS to produce SO_4_^−^·, thus affecting the degradation efficiency of TCH. Based on these findings, it is evident that the reaction system exhibits a broad adaptability to pH variations, enabling a higher TCH removal efficiency without the need for pH adjustment in practical applications.

#### 2.3.4. Effects of NOM

**Figure 10:**
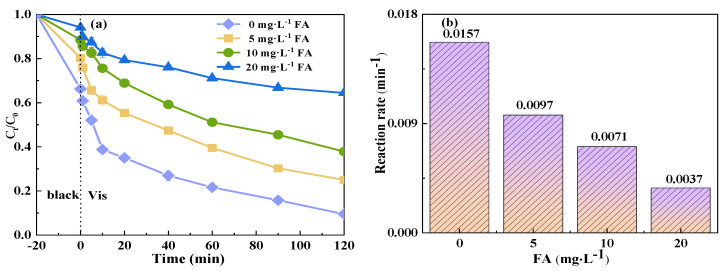
Effect of NOM addition on the degradation of TCH in the Vis+CoFe_2_O_4_/NaBiO_3_+PMS system. (**a**) Degradation efficiency; (**b**) reaction rate. Conditions: [pH] unregulated, [TCH] = 10 mg/L^−1^, [PMS] = 100 mg/L^−1^, [catalyst] = 0.5 g/L^−1^, [FA] = 0, 5, 10, 20 mg/L^−1^.

The composition of wastewater from actual livestock farming is notably complex, containing a significant amount of natural organic matter (NOM), which can affect the oxidation reaction of free radicals. NOM is known to quench free radicals and revert free radical intermediates back into their parent compounds [55]. Consequently, to simulate real-world aquaculture wastewater and examine the influence of organic acids on the degradation of TCH by a photocatalytic system coupled with PMS, FA was introduced. As shown in Figure 10, the addition of FA exerted a discernible inhibitory effect on the catalytic degradation of TCH in the Vis+CoFe_2_O_4_/NaBiO_3_+PMS system. This inhibitory effect became more pronounced with increasing concentrations of FA. Specifically, in the absence of FA (0 mg/L^−1^), the degradation efficiency of TCH reached 90.5%, with a reaction rate of 0.0157 min^−1^. However, both the degradation efficiency and reaction rate decreased gradually with higher FA concentrations. At an FA concentration of 20 mg/L^−1^, the degradation efficiency plummeted to 35.6%, and the reaction rate decreased to 0.0037 min^−1^. This phenomenon can be attributed to the carboxyl and hydroxyl functional groups in FA, which act as free radical scavengers, competing with TCH for active species and thereby impeding TCH degradation [62]. Furthermore, FA can occupy the reaction sites on CoFe_2_O_4_ [63], thereby reducing TCH degradation.

### 2.4. Photocatalytic Mechanism of Vis+CoFe_2_O_4_/NaBiO_3_+PMS System

**Figure 11:**
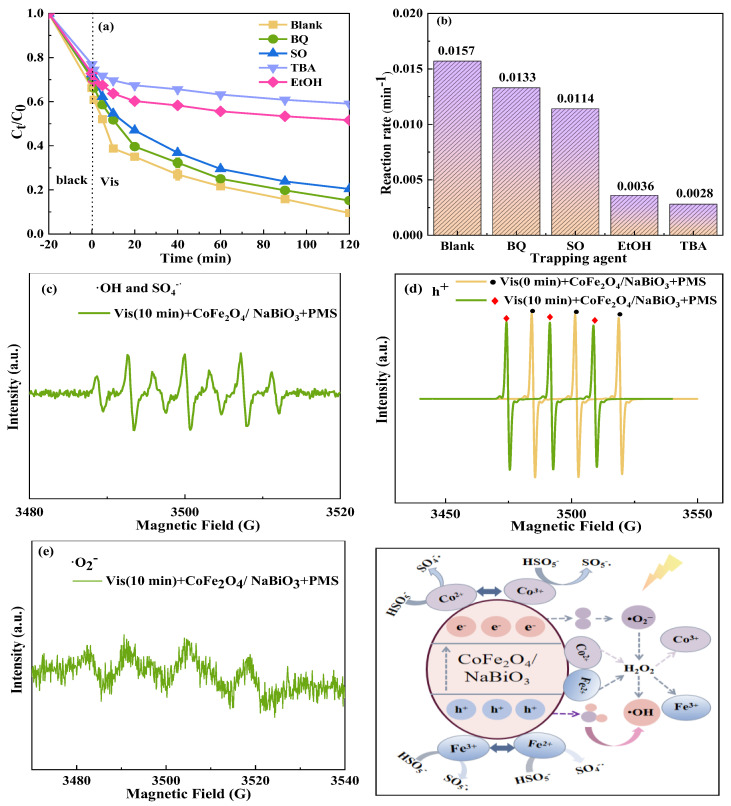
(**a**) The photocatalytic degradation efficiency of TCH with different scavengers. (**b**) Reaction rate; (**c**) DMPO-·OH and DMPO-SO_4_^−^·; (**d**) TEMPO-h^+^; (**e**) DMPO-·O_2_^−^. (**f**) Schematic diagram of degradation mechanism. Conditions: [pH] unregulated, [TCH] = 10 mg/L^−1^, [PMS] = 100 mg/L^−1^, [catalyst] = 0.5 g/L^−1^, [FA] = 0, 5, 10, 20 mg/L^−1^.

To elucidate the primary free radicals generated within the system and clarify the degradation mechanism, various chemical scavengers were introduced into the Vis+CoFe_2_O_4_/NaBiO_3_+PMS system. The findings, presented in Figure 11, indicate that the addition of tertbutyl alcohol (TBA) and ethanol (EtOH) significantly reduced the degradation efficiency from 90.5% to 41% and 48.4%, respectively, and the reaction rate from 0.0157 min^−1^ to 0.0028 min^−1^ and 0.0036 min^−1^. This suggests that ·OH and SO_4_−· are the main active species responsible for TCH degradation in this system. The addition of sodium oxalate (SO) and p-benzoquinone (BQ) resulted in a slight decrease in degradation efficiency by about 11% (79.6%) and 6% (84.8%), respectively, with reaction rates of 0.0114 min^−1^ and 0.0133 min^−1^, indicating that small amounts of h^+^ and ·O_2_^−^ were also produced during the degradation process. The production of ·OH might stem from the photo-Fenton process. The photogenerated electron–hole pair reacts in situ with dissolved oxygen or H_2_O to generate H_2_O_2_ (Equations (4) and (5)) [64]. It, along with Fe^3+^/Fe^2+^, Co^3+^/Co^2+^, constitutes the ideal conditions for the photo-Fenton, leading to the production of ·OH (Equations (6) and (7)) [65]. The formation path of free radicals primarily occurs through interaction of Co^2+^ on the catalyst surface with H_2_O molecules adsorbed on the catalyst, forming CoOH^+^, which further reacts with PMS to form SO_4_−· (Equations (8) and (9)) [66]. SO_4_^−^·can directly degrade the target pollutants and react with H_2_O or OH^−^ to form ·OH (Equations (10) and (11)) [67]. The synergistic effect of CoFe_2_O_4_ and NaBiO_3_ is beneficial to the activation of PMS and promotes the circulation of Co^2+^/Co^3+^ and Fe^3+^/Fe^2+^ valence states to form a dynamic equilibrium (Equations (12)–(16)). Comparative PL tests revealed that CoFe_2_O_4_/NaBiO_3_ broadens the response to visible light and inhibits the recombination of photogenerated electron–hole pairs, facilitating the generation of h^+^, which acts to degrade pollutants, ultimately mineralizing TCH into CO_2_ and H_2_O.

2h^+^ + 2H_2_O → H_2_O_2_ + 2H^+^ (4)

2H^+^ + O_2_+2e^−^ → H_2_O_2_ (5)

Fe^2+^ + H_2_O_2_ → Fe^3+^ +·OH + OH^−^ (6)

Co^2+^ + H_2_O_2_ → Co^3+^ +·OH + OH^−^ (7)

Co^2+^ + H_2_O ↔ CoOH^+^ + H^+^ (8)

CoOH^+^ + HSO_5_^−^ → CoO^+^ + SO_4_^−^·+ H_2_O (9)

SO_4_^−^·+ H_2_O → SO_4_^2−^·+·OH+H^+^ (10)

SO_4_^−^·+ OH^−^ → SO_4_^2−^·+·OH (11)

Co^2+^ + HSO_5_^−^ → Co^3+^ + SO_4_^−^·+ OH^−^ (12)

Co^3+^ + HSO_5_^−^ → Co^2+^ + SO_5_^−^·+ H^+^ (13)

Fe^3+^ + HSO_5_^−^ → Fe^2+^ + SO_5_^−^·+ H^+^ (14)

Fe^2+^ + HSO_5_^−^ → Fe^3+^ + SO_4_^−^·+ OH^−^ (15)

Fe^3+^ + Co^2+^ → Fe^2+^ + Co^3+^ (16)

Electron paramagnetic resonance (EPR) tests further confirmed the active species produced by the Vis+CoFe_2_O_4_/NaBiO_3_+PMS system during TCH degradation (Figure 11c–f). The EPR spectrum showed DMP-OH and DMPO-SO_4_^−^· signal peaks (Figure 11c), attributed to the oxidation of the trapping agent rather than direct signals from OH and SO_4_^−^·, possibly due to the generation of high-valence iron–oxygen species during the redox degradation of pollutants. DMPO was directly oxidized to form a DMPO-X peak shape [68]. The free radical capture test corroborated that ·OH and SO_4_^−^· are produced in the system and play a significant role in pollutant removal. The detection of holes and superoxide radicals under illuminated conditions further supports the system’s capacity to generate these species for pollutant degradation. Figure 11d shows the signal peak of the holes. It was found that the signal intensity generated under no-light conditions was the electronic signal contained in the capturing agent TEMPO. When the signal peak intensity is weakened under light conditions, it proves that the system can generate holes. Figure 11e shows the comparison of DMPO-·O_2_^−^ signal peaks of the system. After illumination for 10 min, the system detected a six-fold signal peak of DMPO-O_2_^−^, indicating that ·O_2_^−^ can be generated in the system during illumination and used for pollutant degradation.

Therefore, through the free radical capture test and EPR test, it was determined that the primary active species in the Vis+CoFe_2_O_4_/NaBiO_3_+PMS system are mainly ·OH > SO_4_^−^·> h^+^ > O_2_^−^.

## 3. Materials and Methods

### 3.1. Reagents

TCH and sodium bismuth dihydrate (NaBiO_3_·2H_2_O) were purchased from Shanghai Yuanye Biotechnology Co., Ltd. (Shanghai, China).; iron nitrate heptahydrate (Fe(NO_3_)_3_·9H_2_O) and cobalt nitrate hexahydrate (Co(NO_3_)_2_·6H_2_O) were purchased from Beijing Minda Technology Co. (Beijing, China); citric acid (C_6_H_8_O_4_), ethylene glycol ((CH_2_OH)_2_), ethanol (C_2_H_5_OH), sodium oxalate (Na_2_C_2_O_4_), p-benzoquinone (C_6_H_4_O_2_), and tert-butyl alcohol (C_4_H_10_O) were purchased from Tianjin Xinbote Chemical Co., Ltd. (Tianjin, China); and potassium monopersulfate (KHSO_5_) was purchased from Shanghai McLean Biochemical Technology Co. (Shanghai, China).

### 3.2. Preparation of CoFe_2_O_4_/NaBiO_3_

CoFe_2_O_4_ was synthesized through the sol–gel method as follows: Firstly, a stoichiometric ratio of 1:2:3 for Fe(NO_3_)_3_·9H_2_O, Co(NO_3_)_2_·6H_2_O, and citric acid was accurately weighed and dissolved in 20 mL of deionized water. This mixture was then stirred at 60 °C for 40 min. Subsequently, 23 mL of ethylene glycol was added to the solution to elevate the temperature to 90 °C. The mixture was continuously stirred until a gel-like resin polymer was formed. This gel was then transferred to a crucible with a lid and placed in a muffle furnace where it underwent calcination at 500 °C for 4 h. Upon cooling, the resultant product was CoFe_2_O_4_.

The preparation of CoFe_2_O_4_/NaBiO_3_ was carried out using solvothermal method: The procedure began with weighing the molar ratio of NaBiO_3_ and CoFe_2_O_4_ at 1:2. The measured CoFe_2_O_4_ was then dispersed into 100 mL of methanol and subjected to ultrasonically dispersed for 20 min to achieve a homogeneous solution. Following this, NaBiO_3_ was introduced into the solution. After stirring for 40 min, the resultant suspension was transferred into a 100 mL Teflon-sealed autoclave and reacted at 180 °C for 10 h. Once the reaction vessel had naturally cooled to room temperature, the obtained products were filtered and subsequently washed 2–3 times using deionized water and anhydrous ethanol, respectively. The final step involved drying the washed products at 60 °C for 12 h in the oven. The dried product was designated as CoFe_2_O_4_/NaBiO_3_.

### 3.3. Characterization of the Catalysts

The surface morphology of CoFe_2_O_4_ and CoFe_2_O_4_/NaBiO_3_ was observed by scanning electron microscopy (SEM; ZEISS Gemini 300, Karl Zeiss, Oberkochen, Germany), with the operating voltage of the morphology at 3 kV. Prior to analysis, all samples underwent a gold sputtering process for 45 s to enhance image clarity. The specific surface area and pore structure characteristics of the catalysts were determined using an Automatic Specific Surface Area and Aperture Distribution Analyzer (BET; AUTOSORB IQ, Quantachrome, Boynton Beac, FL, USA). This analysis involved plotting nitrogen adsorption–desorption isothermal and pore size distribution curves. The chemical composition of CoFe_2_O_4_/NaBiO_3_ was further investigated through X-ray photoelectron spectroscopy (XPS; Thermo Scientific K-Alpha XPS, Thermo Fisher, Waltham, MA, USA). The phase structure of the samples was characterized by X-ray diffraction (XRD; BrukerAXS D8 Advance, Bruker, Karlsruhe, Germany) utilizing a cobalt target over a scanning range of 20–80°and a scanning speed of 2°·min^−1^. An infrared spectrometer (FT-IR; Thermo Scientific Nicolet iS20, Thermo Scientific, Waltham, MA, USA), with a testing wavenumber range of range of 500–4000 cm^−1^, was used to analyze the chemical bonds and functional groups present in the samples. The UV-Vis diffuse reflectance spectra of the samples were measured by a UV-Vis diffuse reflectance spectrometer (UV-Vis DRS; UV-3600i Plus, Shimadzu, Kyoto, Japan) in the wavelength of 200–800 nm. Lastly, the magnetic properties of the catalysts were characterized by a hysteresis loop test (LakeShore 7404, LakeShore, Ouachita Parish, LA, USA).

### 3.4. Photocatalytic Degradation Experiments

To evaluate the catalytic performance of the catalysts, TCH was degraded at its natural pH under visible light irradiation. An initial step involved dispersing 100 mg of photocatalyst in 200 mL of TCH solution (10 mg/L^−1^). The mixture was then stirred in darkness for 20 min using a magnetic rotor stirrer to ensure uniform dispersion, followed by exposure to a 500w xenon lamp (DY500G, Guangzhou Xingtron Electronics Co., Ltd., Guangzhou, China) to simulate visible light degradation. Samples of approximately 5 mL were extracted at predetermined intervals and filtered through a 0.45 μm pore-sized filter membrane, and the absorbance was measured at 356 nm using an ultraviolet spectrophotometer to determine the TCH concentration based on standard calibration curves. The identification of free radicals in the Vis+CoFe_2_O_4_/NaBiO_3_+PMS system was conducted by introducing various quenchers. Investigations into TCH degradation efficiency included varying parameters such as catalyst dosage, PMS concentration, pH levels, initial TCH concentration, and organic matter content. Following adsorption and degradation experiments, the spent catalyst was collected, rinsed with deionized water, and dried under vacuum at 60 °C to assess its stability for subsequent experimental procedures.

## 4. Conclusions

The findings of this study demonstrate that utilizing NaBiO_3_ as a carrier for the synthesis of CoFe_2_O_4_/NaBiO_3_ effectively addresses the issue of CoFe_2_O_4_ agglomeration and concurrently addresses the quandary that NaBiO_3_ is arduous to retrieve. This approach notably enhances the photocatalytic properties of the material by increasing its narrowing the band gap, broadening the absorption of visible light, and significantly reducing the recombination of photogenerated electron–hole pairs, thereby augmenting its photocatalytic efficiency. The coupled Vis+CoFe_2_O/NaBiO_3_+PMS system exhibited a high capability for degrading TCH, achieving a degradation efficiency of 94% after 100 min of illumination under specific conditions: a TCH concentration of 10 mg/L^−1^, a catalyst concentration of 1 g·L^−1^, and a PMS concentration of 100 mg/L^−1^. It was observed that the alkaline environment inhibited the oxidative degradation of TCH, with the highest degradation efficiency occurring under neutral conditions. In addition, the presence of NOM was found to hinder the degradation process. The predominant free radicals contributing to the reaction system were identified as ·OH and SO_4_^−^·. The CoFe_2_O_4_/NaBiO_3_ composite material not only possesses excellent magnetic properties, facilitating easy recovery, but also displays commendable stability, making it a viable option for the treatment of actual wastewater. The primary mechanism underlying the activation of PMS by Vis+CoFe_2_O_4_/NaBiO_3_ involves the valence cycling of Co^3+^ and Co^2+^, as well as between Fe^3+^ and Fe^2+^.

## Data Availability

Data will be made available upon request.
